# Midkine Is a Novel Regulator of Amphetamine-Induced Striatal Gliosis and Cognitive Impairment: Evidence for a Stimulus-Dependent Regulation of Neuroinflammation by Midkine

**DOI:** 10.1155/2016/9894504

**Published:** 2016-12-04

**Authors:** Marta Vicente-Rodríguez, Rosalía Fernández-Calle, Esther Gramage, Carmen Pérez-García, María P. Ramos, Gonzalo Herradón

**Affiliations:** ^1^Pharmacology Lab, Department of Pharmaceutical and Health Sciences, Facultad de Farmacia, Universidad CEU San Pablo, Madrid, Spain; ^2^Biochemistry and Molecular Biology Lab, Department of Chemistry and Biochemistry, Facultad de Farmacia, Universidad CEU San Pablo, Madrid, Spain

## Abstract

Midkine (MK) is a cytokine that modulates amphetamine-induced striatal astrogliosis, suggesting a possible role of MK in neuroinflammation induced by amphetamine. To test this hypothesis, we studied astrogliosis and microglial response induced by amphetamine (10 mg/kg i.p. four times, every 2 h) in different brain areas of MK−/− mice and wild type (WT) mice. We found that amphetamine-induced microgliosis and astrocytosis are enhanced in the striatum of MK−/− mice in a region-specific manner. Surprisingly, LPS-induced astrogliosis in the striatum was blocked in MK−/− mice. Since striatal neuroinflammation induced by amphetamine-type stimulants correlates with the cognitive deficits induced by these drugs, we also tested the long-term effects of periadolescent amphetamine treatment (3 mg/kg i.p. daily for 10 days) in a memory task in MK−/− and WT mice. Significant deficits in the Y-maze test were only observed in amphetamine-pretreated MK−/− mice. The data demonstrate for the first time that MK is a novel modulator of neuroinflammation depending on the inflammatory stimulus and the brain area considered. The data indicate that MK limits amphetamine-induced striatal neuroinflammation. In addition, our data demonstrate that periadolescent amphetamine treatment in mice results in transient disruption of learning and memory processes in absence of endogenous MK.

## 1. Introduction

Drugs of abuse, such as alcohol and amphetamine and its derivatives, induce neuroinflammation [1]. Proliferation of inflammatory cells such as microglia and astrocytes [2] is a signature of neuroinflammation and a hallmark of pathogenesis associated with different events including neurodegeneration [3–5]. Evidence suggests the possibility that neuroinflammatory processes in drug addiction disorders could lead to neurodegeneration in specific brain areas targeted by drugs of abuse. Accordingly, striatal neuroinflammation induced by methamphetamine seems to underlie cognitive deficits, depression, and parkinsonism reported in methamphetamine addicts [6]. In addition, independent studies have demonstrated a highly significant increase in the prevalence of Parkinson's disease (PD) among addicts to amphetamine-type stimulants [7, 8]. Thus, identification and characterization of new genetic factors involved in drug addiction disorders and inflammation are relevant for validation of new biomarkers and for the development of new drugs that could modulate neuroinflammation processes underlying addiction disorders [9].

Midkine (MK) is a cytokine with important functions in peripheral inflammatory processes in different pathological conditions [10, 11]. MK facilitates the migration of macrophages and neutrophils [12–14] and prevents differentiation of regulatory T-cells by inhibiting the development of tolerogenic dendritic cells [15, 16]. In the central nervous system (CNS), the role of MK in inflammation is poorly understood. It has been shown that this cytokine is not involved in the development of astrogliosis or activation of microglia in a spinal cord injury model [17]. However, MK is found highly upregulated in pathologies characterized by inflammation such as cerebral infarction and neurodegenerative diseases [18, 19] and in different brain areas after administration of drugs of abuse [20]. This evidence supports the hypothesis that MK could play an important role in neuroinflammation. To test this hypothesis, we have now confirmed that amphetamine-induced striatal astrogliosis is enhanced in MK knockout (MK−/−) mice [21], and we have tested for the first time striatal microglial response induced by amphetamine in MK−/− and wild type (WT) mice. To test the possibility of a region-specific regulation of astrogliosis by MK, we have also tested the effects of amphetamine in the hippocampus, an area that draws increasing attention as a responsive brain region in psychostimulants actions [22]. We have also investigated if MK regulates glial response depending on the inflammatory stimulus by comparing the effects of lipopolysaccharide (LPS) injection in MK−/− and WT mice. In addition, since early onset of drug abuse causes a wide range of adverse outcomes in adulthood including cognitive deficits [23], which correlates with the capacity of these drugs to induce striatal neuroinflammation [6], we also tested the long-term effects of periadolescent amphetamine treatment in a memory task in MK−/− mice.

## 2. Materials and Methods

### 2.1. Animals

MK−/− mice were kindly provided by Dr. Thomas F. Deuel (The Scripps Research Institute, La Jolla, CA). MK−/− mice were generated as previously described [24]; 8–10-week-old male MK−/− and WT mice on a C57BL/6J background were used for immunohistochemistry studies; 4-week-old male MK−/− and WT mice were used for periadolescent amphetamine treatment prior to behavioral assessment.

The animals used in this study were maintained in accordance with both the ARRIVE guidelines and the European Union Laboratory Animal Care Rules (Directive 2010/63/EU for animal experiments) and the protocols were approved by the Animal Research Committee of USP-CEU.

### 2.2. Histological Studies: Gliosis

#### 2.2.1. Treatments

MK−/− and WT mice received 4 injections (i.p.) of amphetamine (10 mg/kg) or saline (control, 10 mL/kg), allowing between injections a 2-hour interval. This regimen of administration of amphetamine was previously used to observe differences in the striatal astrocytosis induced by this drug in WT and MK−/− mice [21]. Four days after the animals received the first administration of amphetamine or saline (control), mice were euthanized for immunohistochemistry studies as described below.

In the study with LPS, MK−/− and WT mice received a single i.p. injection of LPS (Sigma, Madrid, Spain) (0.5 mg/kg) or saline (control, 10 mL/kg) and were sacrificed 8 h after the treatment. In order to better dissect possible genotypic differences we used a low dose of LPS that was shown to be useful to test neuroinflammation in mice [25].

#### 2.2.2. Immunohistochemistry Analysis

An equal or greater *n* = 4/group was used in all studies. Mice were transcardially perfused with 4%* p-formaldehyde*, and brains were removed and conserved in* p-formaldehyde* for 24 h and transferred to a 30% sucrose solution containing 0.02% sodium azide for storage at 4°C. 30 *µ*m striatal and hippocampal free-floating sections were processed as previously described [21, 26, 27]. Immunohistochemistry studies were performed in one slice per 180 *µ*m (striatum from bregma 1.54 mm to 0.10 mm; hippocampus from bregma −1.06 mm to −2.54 mm).

In order to study astrogliosis, sections were incubated overnight at 4°C with anti-GFAP antibody (Millipore, Madrid, Spain; 1 : 1000) and then for 30 minutes with the appropriate biotinylated secondary antibody (Vector Laboratories, Burlingame, CA, USA; 1 : 5000) in PBS at room temperature; the avidin-biotin reaction was performed using Vectastain Elite ABC peroxidase kit (Vector Laboratories) following the protocol suggested by the manufacturer; immunolabeling was visualized by using 3,3′-diaminobenzidine (DAB). Sections were mounted on gelatin-coated slides, air-dried overnight, dehydrated through graded ethanol, cleared in xylene, and mounted with DPX medium. To study striatal microgliosis, sections were incubated overnight at 4°C with anti-Iba1 antibody (Wako, Osaka, Japan; 1 : 1000), followed by 30 minutes of incubation with Alexa-Fluor-488 secondary antibody (Invitrogen, Waltham, MA USA; 1 : 500). Photomicrographs were captured with a digital camera coupled to an optical microscope (DM5500B, Leica, Solms, Germany). Analysis was performed using ImageJ (NIH), in the three most central slices of each area. GFAP+ astrocytes were counted in 325 × 435 *µ*m standardized areas in the medial striatum as previously described [21, 26] and in the lacunosum moleculare (LMol) located in CA1 area, in the hippocampus. Iba1+ cells were counted in 1100 *µ*m × 1400 *µ*m standardized areas in the striatum.

### 2.3. Behavioral Studies: Y-Maze

Four independent experiments were performed to reach an appropriate number of subjects per experimental group in Y-maze assays. Treatments began during periadolescence (4-week-old mice). Male MK−/− and WT mice were randomly allocated and injected with either amphetamine (3 mg/kg, i.p.) dissolved in saline or saline (10 mL/kg, i.p.), once daily for 10 consecutive days. Six days after the last administration of amphetamine (or saline), behavioral testing started following previously published protocols [28] and leaving appropriate “washing” periods of time between different Y-maze assays.

In order to study recognition processes in response to novelty and working memory in mice, we used the Y-maze test as previously described [29]. Memory was measured with a 60 min intertrial interval (ITI) between acquisition and retrieval. During the first trial (acquisition), the animal is placed in the centre of the maze and allowed to visit for 5 min two arms (“start” and “other” arms) of a Y-maze with three arms each 34 cm long, 8 cm wide, and 14.5 cm high, the third being blocked with a door. During the second trial (retrieval, 5 min), the door is opened, and the animal is free to access all three arms (“start”; “other”; and “novel” arms). During the test, the number of entries into each arm (when a mouse places all four paws into an arm) was recorded. Discrimination of novelty versus familiarity was studied by calculating the preference for the “novel” arm as a discrimination ratio (Novel/[Novel + Other]) for number of arm entries. Scores greater than 0.5 show preference for the “novel” arm indicating the establishment of spatial memory.

Mice (WT: saline, *n* = 15; amphetamine, *n* = 15, and MK−/−: saline, *n* = 9; amphetamine, *n* = 9) were tested in the Y-maze at 6 weeks of age (6 days after last amphetamine administration) and 7 and 9 weeks of age. All the experiments were conducted during the light phase.

### 2.4. Statistical Analysis

Data were analyzed using two-way ANOVA considering genotype and treatment as variants. Relevant differences were analyzed by post hoc comparisons with Bonferroni's post hoc tests. All statistical analyses were performed using GraphPad Prism 6 program (San Diego, CA, USA).

## 3. Results

### 3.1. Amphetamine-Induced Gliosis in the Striatum Is Enhanced in MK−/− Mice

**Figure 1:**
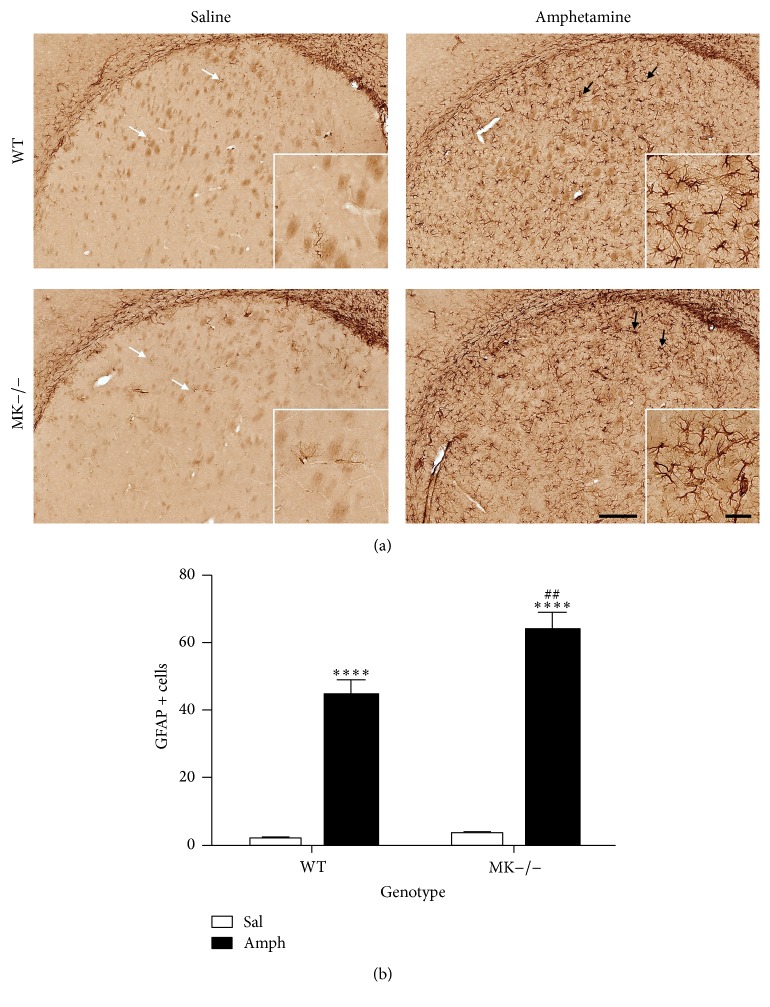
Amphetamine induces astrocytosis in the striatum of WT and MK−/− mice. (a) Photomicrographs are from GFAP-immunostained striatal sections of saline- (Sal-) or amphetamine- (Amph-) treated animals. Amphetamine induced reactive astrocytes characterized by larger densely stained bodies with longer and extensive processes (black arrows) compared to saline-treated mice (white arrows). (b) The graph represents quantification of data (mean ± SEM) obtained from the counts of GFAP-positive cells in standardized areas of the striatum. Significant effects of the genotype (*F*_(1,16)_ = 8.051, *P* = 0.01), the treatment (*F*_(1,16)_ = 200.3, *P* < 0.0001), and genotype by treatment variant interaction (*F*_(1,16)_ = 5.896, *P* = 0.03) were found. ^*∗∗∗∗*^ *P* < 0.0001 versus Sal. ^##^ *P* < 0.01 versus WT. Scale bar = 200 *μ*m. Magnified inset = 50 *μ*m.

In order to confirm the previously reported enhanced astrocytosis induced by amphetamine in MK−/− mice [21], immunohistochemical analysis of GFAP-positive cells in the striata of MK−/− and WT mice was performed. Compared to saline-treated animals from both genotypes, amphetamine induced reactive astrocytes characterized by large densely stained bodies with longer and extensive processes (Figure 1(a), black arrows). As expected, amphetamine efficiently increased the number of GFAP+ cells in both genotypes (Figure 1(b)). The number of GFAP+ astrocytes in the striata of amphetamine-treated MK−/− mice was significantly higher than that in WT mice (Figure 1(b)). The data confirm that MK regulates amphetamine-induced striatal astrocytic response.

**Figure 2:**
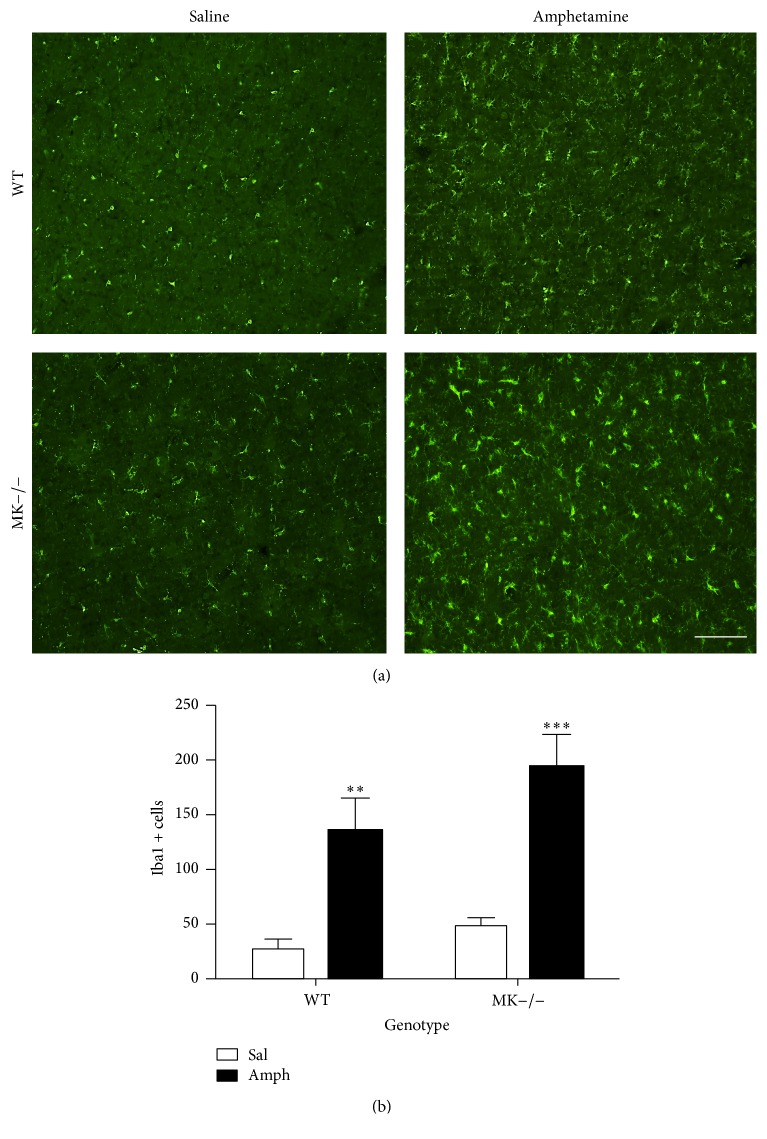
Amphetamine induces microgliosis in the striatum of WT and MK−/− mice. (a) Photomicrographs are from Iba-1-immunostained striatal sections of saline- (Sal-) or amphetamine- (Amph-) treated animals. (b) The graph represents quantification of data (mean ± SEM) obtained from the counts of Iba-1-positive cells in standardized areas of the striatum. Significant effects of the genotype (*F*_(1,10)_ = 4.597, *P* = 0.05) and treatment (*F*_(1,10)_ = 46.12, *P* < 0.0001) were found. ^*∗∗*^ *P* < 0.01 and ^*∗∗∗*^ *P* < 0.001 versus Sal. Scale bar = 200 *μ*m.

Augmented microglial response is a hallmark of amphetamine derivative-induced neurotoxicity [30] and a signature of neuroinflammation. Immunohistochemistry for Iba1 revealed a significant increase in the number of Iba1+ cells in the striata of WT mice 4 days after amphetamine treatment (Figure 2). Compared to saline-treated mice, we found a more pronounced increase in Iba1-ir following amphetamine administration to MK−/− mice (Figure 2). The data demonstrate for the first time that MK modulates the microglial response induced by amphetamine.

### 3.2. Genetic Inactivation of Midkine Prevents Amphetamine-Induced Astrogliosis in Hippocampus

**Figure 3:**
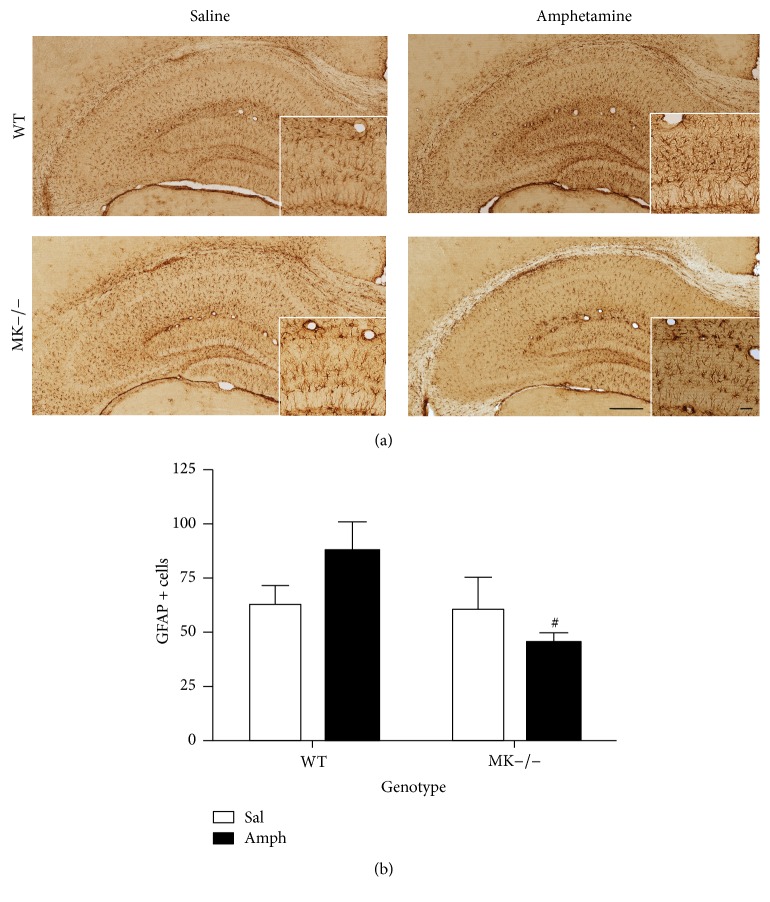
Amphetamine-induced astrocytosis in the hippocampus of WT and MK−/− mice. (a) Photomicrographs are from GFAP-immunostained hippocampal sections of saline- (Sal-) or amphetamine- (Amph-) treated animals. (b) The graph represents quantification of data (mean ± SEM) obtained from the counts of GFAP-positive cells in standardized areas of the CA1 region of hippocampus. A significant effect of the genotype (*F*_(1,12)_ = 4.88, *P* = 0.04) was found. ^#^ *P* < 0.05 versus WT. Scale bar = 200 *μ*m. Magnified inset = 50 *μ*m.

The data obtained in the striatum of WT and MK−/− mice suggest that MK regulates the glial response induced by amphetamine. The data are particularly robust in the case of the astrocytic response which is highly increased after amphetamine treatment in MK−/− mice. To test the possibility that MK regulates astrocytosis depending on the brain area considered, we also assessed the effects of amphetamine in the hippocampus of MK−/− and WT mice (Figure 3(a)). Amphetamine induced different effects in WT and MK−/− mice (Figure 3). In contrast to the astrocytic response in the striatum, a significant decrease of GFAP+ astrocytes in amphetamine-treated MK−/− mice compared to WT mice (Figure 3(b)) was observed. The data demonstrate a regional-specific regulation of amphetamine-induced astrocytosis by MK.

### 3.3. Genetic Inactivation of Midkine Differentially Regulates LPS-Induced Striatal Gliosis

**Figure 4:**
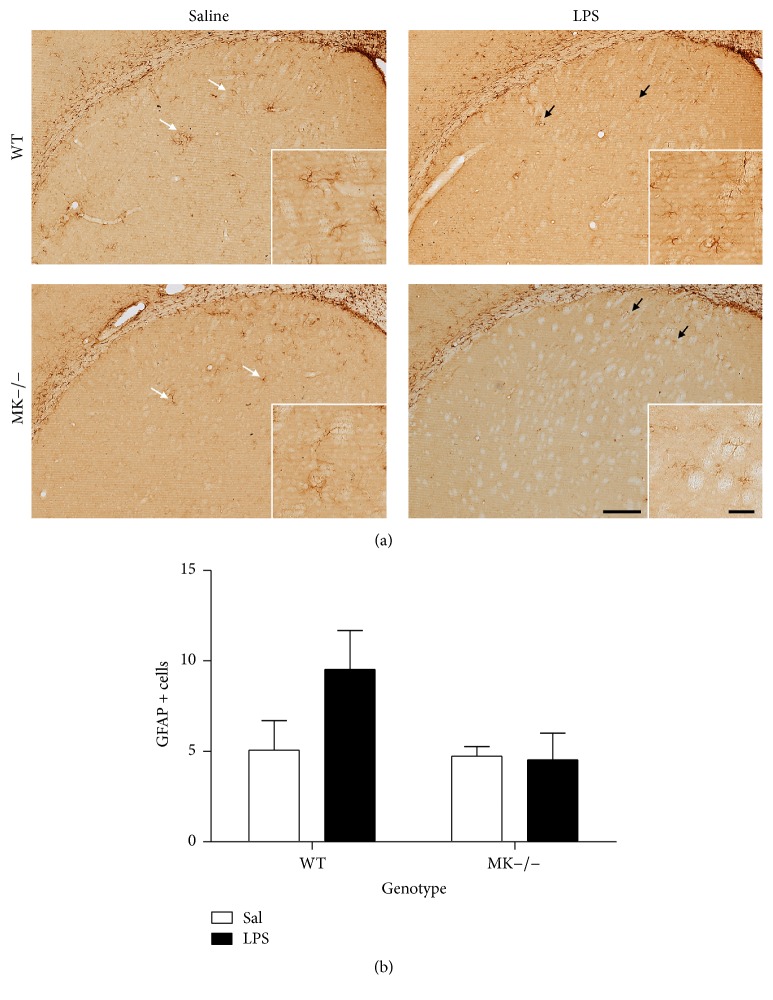
LPS-induced astrocytosis in the striatum of WT and MK−/− mice. (a) Photomicrographs are from GFAP-immunostained striatal sections of saline- (Sal-) treated or 0.5 mg/kg lipopolysaccharide- (LPS-) treated animals. (b) The graph represents quantification of data (mean ± SEM) obtained from the counts of GFAP-positive cells in standardized areas of the striatum. Scale bar = 200 *μ*m. Magnified inset = 50 *μ*m.

**Figure 5:**
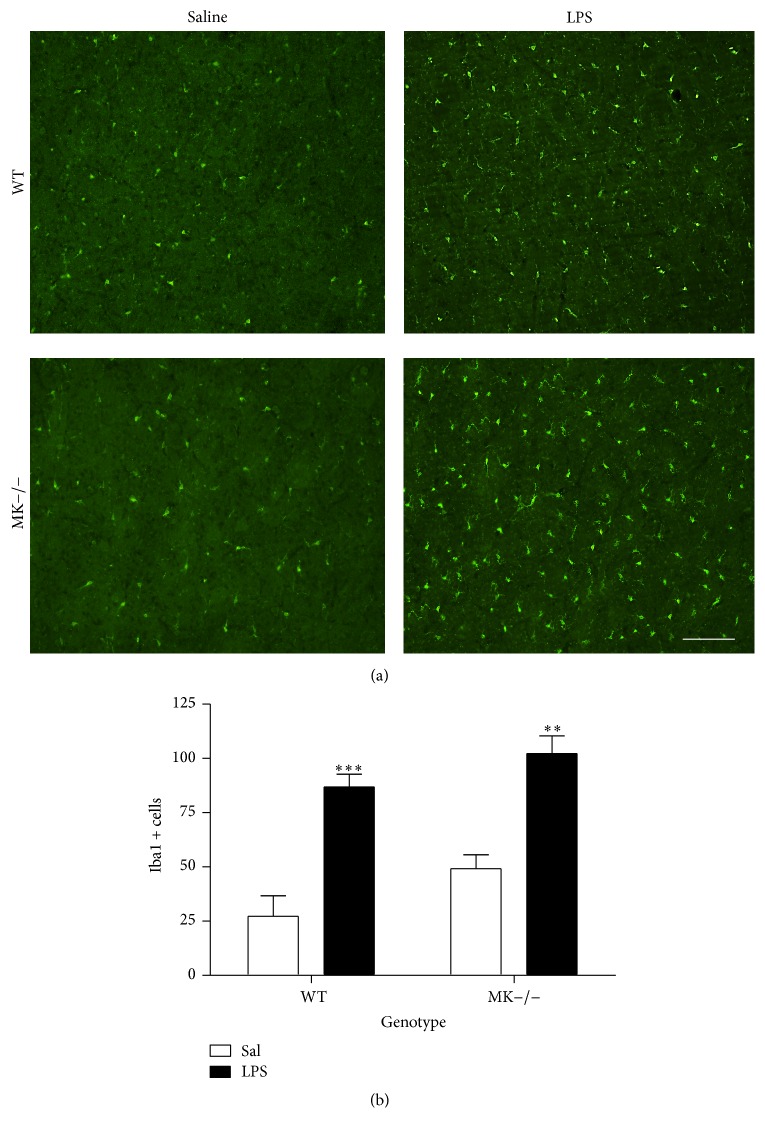
LPS-induced microgliosis in the striatum of WT and MK−/− mice. (a) Photomicrographs are from Iba-1-immunostained striatal sections of saline- (Sal-) treated or 0.5 mg/kg lipopolysaccharide- (LPS-) treated animals. (b) The graph represents quantification of data (mean ± SEM) obtained from the counts of Iba-1-positive cells in standardized areas of the striatum. Significant effects of the genotype (*F*_(1,12)_ = 5.17, *P* = 0.04) and treatment (*F*_(1,12)_ = 47.41, *P* < 0.0001) were found. ^*∗∗*^ *P* < 0.01 and ^*∗∗∗*^ *P* < 0.001 versus Sal. Scale bar = 200 *μ*m.

MK is not only found upregulated in the brain after administration of different drugs of abuse. The cerebral levels of expression of MK are increased in different diseases that share neuroinflammation as a common pathogenic mechanism, including ischemia and neurodegenerative diseases [20]. To test the possibility that MK regulates the glial response depending on the inflammatory stimulus used, we assessed the effects of LPS in MK−/− and WT mice. As expected, a low dose of LPS (0.5 mg/kg) induced a moderate ~2-fold increase in the number of GFAP+ cells in the striatum of WT mice, an effect that was completely absent in MK−/− mice (Figure 4). In the case of microglia, LPS induced a robust response in both genotypes (Figure 5). However, compared to their corresponding saline-paired control groups, the increases in the number of Iba-1+ cells in the striatum after LPS administration tended to be similar in WT and MK−/− mice (Figure 5(b)). The data demonstrate that MK modulates the astrocytic, but not microglial, response induced by LPS in the striatum.

### 3.4. Effect of Amphetamine on Y-Maze Behavior

**Figure 6:**
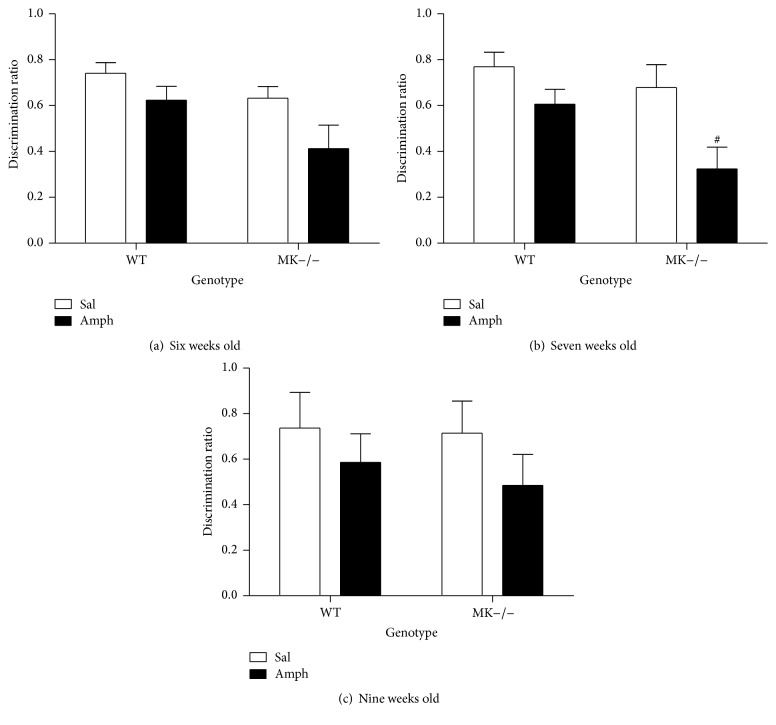
Behavioral performance in the Y-maze of WT and MK−/− mice treated with amphetamine during adolescence. (a) Figure shows mean ± SEM of discrimination ratio for 6-week-old WT and MK−/− mice pretreated with amphetamine during adolescence. Significant effects of the genotype (*F*_(1,44)_ = 5.04, *P* = 0.03) and the treatment (*F*_(1,44)_ = 6.03, *P* = 0.02) were found. (b) Figure shows mean ± SEM of discrimination ratio for 7-week-old WT and MK−/− mice pretreated with amphetamine. Significant effects of the genotype (*F*_(1,44)_ = 5.1, *P* = 0.03) and treatment (*F*_(1,44)_ = 10.46, *P* = 0.002) were found. (c) Figure shows mean ± SEM of discrimination ratio for 9-week-old WT and MK−/− mice pretreated with amphetamine. ^#^ *P* < 0.05 versus WT.

The data presented here demonstrate for the first time an important role of MK in the modulation of the neuroinflammatory processes induced by amphetamine in the striatum. These processes have been related to the cognitive deficits caused by amphetamines abuse in humans [6]. Thus, we next tested the possibility that endogenous MK modulates the cognitive effects caused by a 10-day amphetamine treatment during adolescence, a stage especially vulnerable to the cognitive deficits induced by these drugs [23, 31]. Six days after the last amphetamine (or saline) administration, we assessed for the first time the behavior of six-week-old mice from both genotypes on the Y-maze (Figure 6). To test the possible long-term effects of amphetamine treatment during adolescence on the behavioral performance of WT and MK−/− mice on a memory task, we tested them again one week (7-week-old mice) and three weeks later (9-week-old mice). First, it was found that control (saline-treated) WT and MK−/− mice efficiently established spatial memory in a similar manner (Figures 6(a)–6(c)). Amphetamine treatment during adolescence tended to impair the recognition memory in the Y-maze in 6-week-old MK−/− mice (Figure 6(a)). This amphetamine-induced impairment of the recognition memory was exacerbated in 7-week-old MK−/− compared to WT mice (Figure 6(b)). However, this effect of amphetamine in recognition memory of 6- and 7-week-old MK−/− mice was found to be transient since it was abolished in 9-week-old mice (Figure 6(c)). The data indicate that the cognitive impairment caused by periadolescent amphetamine treatment is modulated by endogenous MK.

## 4. Discussion

According to the European Monitoring Centre for Drugs and Drug Addiction, ever in lifetime use of amphetamines among young people in Europe varies considerably, with levels of 30–70%. Despite widespread use of amphetamine-type stimulants, the medical consequences of these drugs abuse and the mechanisms underlying them are only partially understood. These drugs cause neuroinflammation [1], a pathogenic mechanism contributing to amphetamine-induced dopaminergic injury in the nigrostriatal pathway [32]. Accordingly, significant increases in the prevalence of PD in amphetamine-type drug abusers have been reported [7, 8]. In the present work, we have confirmed that amphetamine-induced astrogliosis in the striatum, a hallmark of amphetamine-type drugs-induced neuroinflammation, is potentiated by genetic inactivation of MK [21]. More importantly, we demonstrate for the first time that amphetamine-induced striatal microglial response is also enhanced in MK−/− mice. The data indicate that MK is a genetic factor that regulates the neuroinflammatory effects induced by this type of psychostimulants. In this context, it is also important to note that MK expression and signaling are activated in the brain after administration of different drugs of abuse [33–35]. Taking together, our data suggest that the previously shown neuroprotective effects of MK against drug-induced neurotoxicity [20, 36] could be related to its ability to prevent neuroinflammation.

The counteractive effect of MK against amphetamine astrogliosis seems to be region-specific since it is observed in the striatum, the main area affected by the neurotoxic effects of amphetamine, but not in the hippocampus. One possible explanation for these differences could be related to the different pattern of expression of MK after injury in both areas. While increased expression of MK is found in GFAP+ astrocytes in the injured mouse hippocampus [37], MK expression in the neurodegenerative nigrostriatal pathway is mainly found in neurons [20]. Thus, it is reasonable to hypothesize that constitutive genetic deletion of MK could cause different effects in response to injury depending on the area considered.

Midkine expression levels in the brain are also upregulated in different pathologies characterized by overt neuroinflammation [3, 10, 20, 38]. Midkine is known to exert neuroprotective effects in some of these pathologies including Alzheimer's disease [18] and brain ischemia [39]. Thus, it is reasonable to hypothesize that the ability of MK to limit neuroinflammation could contribute to its neuroprotective actions in different pathological contexts. However, our data indicate that microglial response after LPS administration is not significantly regulated by MK. In contrast, LPS-induced striatal astrogliosis was blocked by genetic inactivation of MK. The data demonstrate a differential regulation of astrocytosis by MK depending on the inflammatory stimulus. The data presented here provide novel insights in the mechanisms and roles played by MK in CNS disorders in which astrogliosis is known to play pivotal roles. For instance, MK is expressed in senile plaques of Alzheimer's disease patients [40]. Astrocytosis facilitates A*β* plaque deposition in Alzheimer's disease [41] suggesting the interesting possibility that MK decreases A*β* plaque deposition [18] through its ability to prevent astrogliosis.

The mechanism of action of MK supports our findings. One receptor for MK is Receptor Protein Tyrosine Phosphatase *β*/*ζ* (RPTP*β*/*ζ*) (a.k.a. PTPRZ1) [42]. MK binds to RPTP*β*/*ζ* and inactivates its phosphatase activity. Inhibition of the phosphatase activity of RPTP*β*/*ζ* by MK binding regulates the tyrosine phosphorylation levels of substrates of RPTP*β*/*ζ* which are known regulators of neuroinflammatory processes such as TrkA [43]. Signaling pathways downstream of MK/RPTP*β*/*ζ* which are also known to participate in gliosis include MAPK pathways [10]. Further studies are needed to test the possible involvement of these signaling pathways in the modulatory actions of MK in neuroinflammation.

Overall, the data presented here are relevant because the roles of MK in promoting inflammation had been described in detail in peripheral organs such as kidney and liver [10] but little was known about a possible role of MK in central inflammation. We now demonstrate for the first time that MK is a novel modulator of amphetamine-induced neuroinflammation in a brain area-dependent manner. We also show that MK differentially regulates astrogliosis depending on the noxious stimulus that triggers neuroinflammatory processes. Our data support the need of further studies to dissect the specific modulatory roles of MK on neuroinflammation in the different brain pathologies in which MK has been shown to be upregulated.

In addition to its involvement in neurotoxicity and neurodegeneration, neuroinflammation significantly contributes to behavioral alterations possibly by affecting synaptic function [44]. Accordingly, new experimental therapeutics provide correlation between reduction of neuroinflammation and improved cognitive impairment in Alzheimer's disease [45]. Interestingly, behavioral changes associated with amphetamine treatment during adolescence are cognitive deficits [31]. Our data demonstrate that periadolescent amphetamine treatment has modest consequences in WT mice in the Y-maze test but causes a transient disruption of the working memory in the Y-maze in MK−/− mice. The data suggest that genetic inactivation of MK confers greater vulnerability to the transient cognitive impairment associated with a periadolescent amphetamine treatment. It is tempting to connect these behavioral data with the reduced amphetamine-induced astrocytosis in the MK−/− mouse hippocampus, a relevant area for recognition memory. However, in the case of this type of drugs, it has been recently shown that neuroinflammatory processes induced by methamphetamine in the striatum underlie cognitive deficits in this drug's addicts [6]. Our data support the correlation between cognitive deficits and enhanced amphetamine-induced striatal neuroinflammation in MK−/− mice.

## 5. Conclusions

The data demonstrate for the first time that MK is a novel regulator of neuroinflammation in a stimulus and brain region-dependent manner. The data presented here indicate that MK limits amphetamine-induced striatal neuroinflammation. The data also demonstrate that periadolescent amphetamine treatment in mice results in transient disruption of learning and memory processes in MK−/− mice, effect that could be related to the absence of the counteractive actions of MK in striatal neuroinflammation.
